# Di2-ethylhexyl phthalate disrupts thyroid hormone homeostasis through activating the Ras/Akt/TRHr pathway and inducing hepatic enzymes

**DOI:** 10.1038/srep40153

**Published:** 2017-01-09

**Authors:** Hanfeng Ye, Mei Ha, Min Yang, Ping Yue, Zhengyuan Xie, Changjiang Liu

**Affiliations:** 1 Yunnan Key Lab of Fertility Regulation and Minority Birth Health, Yunnan Population and Family Planning Science and Technology Research Institute, Yunnan 650021, P.R. China; 2 School of Nursing, Chongqing Medical and Pharmaceutical College, Chongqing 400020, P.R. China; 3 Key Lab of Birth Defects and Reproductive Health of National Health and Family Planning Commission, Chongqing Population and Family Planning Science and Technology Research Institute, Chongqing 400020, P.R. China

## Abstract

Di(2-ethylhexyl) phthalate (DEHP), as a widespread environmental pollutant and an endocrine disruptor, can disturb the homeostasis of thyroid hormones (THs). In order to elucidate roles of the MAPK and PI3K/Akt pathways and hepatic enzymes in thyroid-disrupting effects of DEHP, Sprague-Dawley rats were dosed with DEHP by gavage for 30 consecutive days; Nthy-ori 3-1 cells were treated with DEHP with NAC, k-Ras siRNA or inhibitors (U0126 and wortmannin). Results showed that DEHP led to histopathologic changes in rat thyroid and liver, such as the decrease in thyroid follicular cavity diameter, hepatocyte edema. Triiodothyronine (T3), thyroxine (T4) and thyrotropin releasing hormone (TRH) were reduced. DEHP caused ROS production, oxidative stress and k-Ras upregulation, thereby activating the ERK and Akt pathways *in vivo* and *in vitro*. Moreover, TRH receptor (TRHr) level was elevated after the activation of the Akt pathway and was downregulated after the inhibition of the Akt pathway. However, TRHr was not modulated by the ERK pathway. Additionally, hepatic enzymes, including Ugt1a1, CYP2b1, Sult1e1, and Sult2b1, were significantly induced after DEHP exposure. Taken together, DEHP can perturb TH homeostasis and reduce TH levels. The activated Ras/Akt/TRHr pathway and induced hepatic enzymes play vital roles in thyroid-disrupting effects of DEHP.

Thyroid hormones (THs) that are synthesized and released by the thyroid play important roles in many physiological systems, such as development and differentiation of organs, metabolism of organisms, and energy homeostasis, and alterations in TH levels would result in a series of adverse subclinical or clinical conditions. Especially for children, the disturbance of TH homeostasis during the development of the nervous system would lead to irreversible mental retardation^1^. Therefore, the TH-disrupting factor is the subject of intensive research. In recent years, phthalate esters have attracted considerable attention of the scientific community and the general public, since some evidence has suggested that the thyroid is vulnerable to endocrine-disrupting effects of phthalate esters^2^,^3^.

Di2–ethylhexyl phthalate (DEHP), the most common phthalate ester, is widely used in various consumer products such as food packaging, medical devices and toys, to impart flexibility and durability to polyvinyl chloride based plastics. DEHP is not covalently bound to plastic matrix and can be released from the substrates into the environment. As a widespread environmental pollutant and an endocrine disruptor, DEHP has been paid extensive concern for its potential toxic effects such as reproductive toxicity^4^, neurotoxicity^5^, hepatotoxicity^6^, and carcinogenicity^7^. In addition, DEHP is also reported to have thyroid-disrupting effects on humans and animals. The study by Huang *et al*.^8^ found that the urinary MBP (a metabolite of DEHP) level was negatively associated with TT4 and FT4 in pregnant women. When SD rats were treated with DEHP (1000 mg/kg) by gavage for 10 days, serum TT4 level was reduced by 25.0%^9^.

As relatively more studies have been focusing on the disruptive effect of phthalates on thyroid function and TH homeostasis, some potential mechanisms have been suggested. In zebrafish embryos, MEHP (the main metabolite of DEHP) reduced TT3 and TT4 levels via upregulating gene expressions of type I and II deiodinase, sodium/iodide symporter (NIS), thyroglobulin, and Ugt1ab^10^. A study in a rat FRTL-5 cells also reported that DEHP treatment caused changes in iodide uptake of thyroid follicular cells^11^. Another mechanism proposed is associated with TH receptors. Phthalates could bind to TH receptors (TRα and TRβ) to disturb the signaling of THs and then affect TH production^12^. In hyperthyroid rats, TRα1 expression was elevated after exposure to di(n-butyl) phthalate^13^. Moreover, Ishihara *et al*.^14^ observed that phthalates could competitively bind to transthyretin (TTR), a major TH-binding transport protein. Our previous study also found that DEHP suppressed gene and protein expression of TTR in rat liver, contributing to the decrease in circulating TH levels in serum^15^.

Thyroid hormone synthesis and secretion are exquisitely modulated by a negative feedback system of the hypothalamic-pituitary-thyroid (HPT) axis, and the transduction of TH signaling is closely associated with multiple signaling pathways, such as the phosphoinositide 3-kinase (PI3K)/Akt and mitogen-activated protein kinase (MAPK) pathways. The study by Serrano-Nascimento *et al*.^16^ suggested that the involvement of the PI3K/Akt pathway was a novel mediator of the I^−^-induced thyroid autoregulation. Our previous study also observed that the activated JNK/MAPK pathway played key roles in PCB153-mediated TH reduction in serum^17^. However, there are very few studies that have examined roles of signaling pathways in thyroid-disrupting effects of phthalates.

Hereon, we hypothesize that signaling pathways such as the MAPK and PI3K/Akt pathways may function in the phthalates-mediated disruption of TH homeostasis. To verify the hypothesis, SD rats and human thyroid follicular epithelial cell line were used in the present study. Our findings demonstrate that the Ras/Akt pathways play critical roles in DEHP-caused TH reduction via TRHr. In addition, our study also indicates that the induction of hepatic enzymes by DEHP contributes to the decline of THs.

## Materials and Methods

### Animals and treatments

Twenty-four male Sprague-Dawley rats 18 days old, weighting 41.7 ± 6.4 g, were purchased from the Daping Hospital Animal Laboratory (Chongqing, China) and allowed to acclimatize for one week. Animals were housed at 22 °C with a 12-h light/dark cycle and divided into four groups (n = 6/dose group). Rats had free access to food and water. Animals were treated with DEHP or corn oil (vehicle control) by gavage for 30 consecutive days and the doses were 250, 500, and 750 mg/kg. The age of animals and duration of exposure used were based on the recommendations of the US EPA Endocrine Disrupter Screening and Testing Advisory Committee. The dose (750 mg/kg) that could cause adverse effects without inducing systematic toxicity was utilized according to the lowest observed effect level (LOEL) in pubertal rats^18^ and our previous observation^4^. After ethylether anesthesia, treated rats were sacrificed within 24 h after the last dose. All protocols for animal use conformed to the Guide for the Care and Use of Laboratory Animals established by the MOH of China. All experimental protocols were approved by the Research Ethics Committee of Chongqing Population and Family Planning Science and Technology Research Institute.

### Cell culture and treatments

The human thyroid follicular epithelial cell line (Nthy-ori 3-1) was obtained from the European Collection of Cell Cultures (Salisbury, UK). Nthy-ori 3-1 cells were maintained in RPMI 1640 medium with 10% fetal bovine serum in a humidified incubator (37 °C, 5% CO_2_). NAC was dissolved in sterile PBS and diluted to 1 mM. U0126 and wortmannin (Wort.) were prepared in DMSO and diluted to 50 μM and 20 μM, respectively. After 1-h pretreatments with NAC or inhibitors, cells were treated with DEHP for different time.

### Cytotoxicity assay

Cell viability was measured by MTT assay. In brief, Cells were treated with various concentrations of DEHP in 96-well plates for 24 h, followed by treatment with MTT solution (20 μl, 5 mg/ml) at 37 °C for 4 h. After the removal of medium, DMSO (200 μl) was added to dissolve the blue MTT formazan crystal, and optical intensity was assayed at 570 nm.

### k-Ras gene silencing

After grown to 60–80% confluent, Nthy-ori 3-1 cells were transfected with the siRNA (k-Ras, ID: s7940). The validated siRNA was obtained from the Life Technologies Corporation to specifically silence k-Ras gene of cells. The siRNA was resuspended in nuclease-free sterile water and diluted in Opti-MEM Medium to 200 nM. The siRNA-Lipofectamine^®^ RNAiMAX complex was prepared according to the protocol. Then transfected cells were incubated at 37 °C for 24 h.

### Hormone measurement

Serum levels of TT4, TT3, FT4, FT3, TSH and TRH were determined in duplicate using enzyme-linked immunoabsorbent assay (ELISA) kits (EIAab Science Co. Ltd., China). Details on standard curve concentrations for each ELISA kit are available from the manufacturer.

### Histopathologic evaluation

#### Histological evaluation

The thyroid and liver were fixed in 4% paraformaldehyde for 24 h at room temperature. Then, the thyroid and liver were embedded in paraffin and sliced. Sections were stained with hematoxylin and eosin for microscopic examinations. The image analysis software, Image-Pro Plus 6.0, was applied to quantitatively analyzed histological changes of thyroids and livers, including the number of thyroid follicular epithelial cells and hepatocytes, thyroid follicular cavity diameter, as well as hepatocyte diameter.

### Ultrastructural assessment

The thyroid was fixed in 2.5% glutaraldehyde at 4 °C for 24 h. One percent osmium tetroxide was sued to postfix the slices of thyroids. Sections were analyzed with FEI Tecnai 12G^2^ transmission electron microscope.

### Oxidative stress and ROS analysis

#### Oxidative stress assay

After preparations of liver homogenate, DEHP-caused oxidative stress in the rat liver was determined using the GSH-Px, SOD and MDA Assay Kits according to manufacturer’s protocols (Jiancheng Bioengineering Ltd., China). All samples were detected in duplicate.

### Dichlorofluorescein (DCF) assay for ROS

DEHP-induced ROS production in cells was evaluated utilizing the oxidant-sensitive probe DCFH-DA. Nthy-ori 3-1 cells were treated with DEHP (400 μM) for 24 h in the presence or absence of NAC (1 mM). And then, cells were rinsed and incubated with DCFH-DA (10 μM) at 37 °C for 20 min. Cells were resuspended and seeded in 96-well plates after the trypsinization and collection. The fluorescence intensity of treated cells was quantitatively analyzed by a fluorescent microplate reader (excitation wavelength, 488 nm; emission wavelength 525 nm).

### Real-time PCR analysis

Real-time PCR (RT-PCR) was performed with an ABI PRISM^®^ 7900HT Sequence Detection System (Applied Biosystems, USA) after preparations of the total RNA and cDNA. Thermal cycling conditions comprised two initial steps at 50 °C for 2 min and 95 °C for 2 min, followed by 40 cycles at 95 °C for 15 sec and at 60 °C for 1 min. The primer sequences used were from the GenBank (Table 1). β-Actin was used in parallel for each run as internal control. Each sample was processed in triplicate.

### Western blot determination

Proteins of rat tissues (hypothalamus and liver) and Nthy-ori 3-1 cells were extracted using the Cell Lysis Buffer for Western Blot and IP. Equal amounts (20 μg/lane) of protein were separated by SDS-PAGE and transferred electrophoretically onto a nitrocellulose membrane. Then the membrane was blocked in blocking buffer for 1 h at room temperature. Membranes were incubated with primary antibodies overnight at 4 °C, followed by the treatment with corresponding secondary antibodies at 37 °C for 1 h. The ECL system was utilized to visualize specific proteins.

### Immunohistochemical (IHC) staining analysis

Specimens of thyroid and liver were fixed in 4% paraformaldehyde, embedded in paraffin and sliced to a thickness of 4 μm. After a series of conventional procedures, the sections were incubated with anti-p-Akt or anti-Sult1e1 overnight at 4 °C. Then, the sections were treated with secondary antibodies, peroxidase-conjugated streptavidin, diaminobenzidine substrate, and hematoxylin the next day.

### Immunofluorescent (IF) staining analysis

After a series of conventional operations, the sections of thyroid and liver were incubated with anti-p-ERK or anti-CYP2b1 overnight at 4 °C. Then, the sections were treated with secondary antibodies, DAPI, and antifade polyvinylpyrrolidone mounting medium the next day.

### Confocal laser scanning microscopy (CLSM) analysis

After fixed with 4% paraformaldehyde and treated with goat serum, Nthy-ori 3-1 cells were incubated with anti-TRHr overnight at 4 °C. Nuclei were stained with DAPI (10 μg/ml) for 10 min after treatment with the secondary antibody. Cells were washed and mounted with antifade polyvinylpyrrolidone mounting medium for CLSM analysis.

### Statistical analysis

Values are reported as mean ± SD. Statistical analyses of the results were performed by one-way ANOVA. Mean values were compared by least-significant difference (LSD) using the SPSS. Differences were considered significant when *P* < 0.05.

## Results

### DEHP increased the liver weight of rats

No lethality or obvious signs of toxic effects were observed and all animals had normal activities during the course of exposure. Exposure to DEHP did not lead to changes of final body weight and body weight gain. However, significant increases in liver weight (*P* = 0.013) and liver/body weight ratio (*P* < 0.001) were observed following treatment with DEHP, relative to the control (Table 2).

### DEHP decreased hormone levels

**Figure 1:**
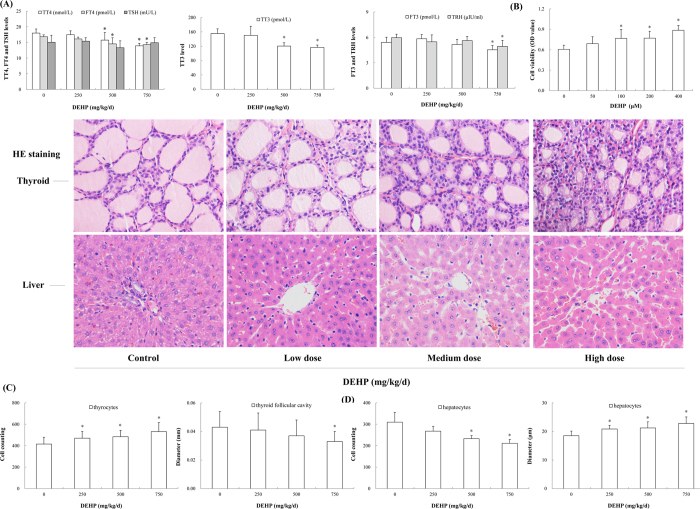
Effects of DEHP on serum thyroid hormones, cell viability and morphology of rat thyroid and liver. Rats were treated daily with DEHP (0, 250, 500, 750 mg/kg) by gavage for 30 days. Histological alterations in the thyroid and liver were evaluated by HE staining. Magnification: X400. (**A**) Serum TT4, FT4, TT3, FT3 and TRH levels were reduced, whereas TSH level was not affected after DEHP exposure. (**B**) The viability of Nthy-ori 3-1 cells was stimulated following DEHP treatment. (**C**) Histological changes in the thyroid were observed, as characterized by increased numbers of thyroid follicular epithelial cells and decreased thyroid follicular cavity diameter. (**D**) Histological changes in the liver were observed, as characterized by reduced hepatocyte number and elevated hepatocyte diameter. Error bars represent the standard deviation. *P < 0.05, compared with the control.

Circulating TH levels in serum were significantly reduced after exposure to DEHP, with maximum effects observed in the high-dose group. Compared with the control, levels of TT4, FT4, TT3 and FT3 in the high-dose group were decreased by 22.6% (*P* < 0.001), 15.1% (*P* = 0.001), 24.7% (*P* < 0.001) and 15.9% (*P* = 0.012), respectively. Likewise, a significant decreasing trend was observed in TRH level. In the high-dose group, TRH level was downregulated to 82.9% of the control (*P* = 0.009). However, DEHP exposure did not significantly affect TSH level (Fig. 1A).

### DEHP stimulated the viability of thyrocytes

DEHP had stimulatory effects on Nthy-ori 3-1 cells, a concentration-dependent increase in the cell viability being observed. Especially in the 400 μM group, the viability of thyrocytes was elevated by 46.0% over the control (Fig. 1B; *P* < 0.001).

### DEHP led to histopathologic changes

HE staining analysis revealed exposure-related alterations in rat thyroid and liver. The thyroid of treated animals exhibited signs for hyperactivity, as indicated by an increase in the number of thyroid follicular epithelial cells (*P* < 0.001) and a decrease in thyroid follicular cavity size (Fig. 1C; *P* = 0.015). Moreover, the cytoplasm that appeared foamy and vacuolated was also observed in the enlarged follicular epithelial cells. Furthermore, DEHP caused histological changes in the liver, such as vacuolation, hepatic sinusoidal dilation. Hepatocyte edema was also observed and characterized by reduced hepatocyte number (*P* = 0.006) and elevated hepatocyte diameter (Fig. 1D; *P* < 0.001).

**Figure 2:**
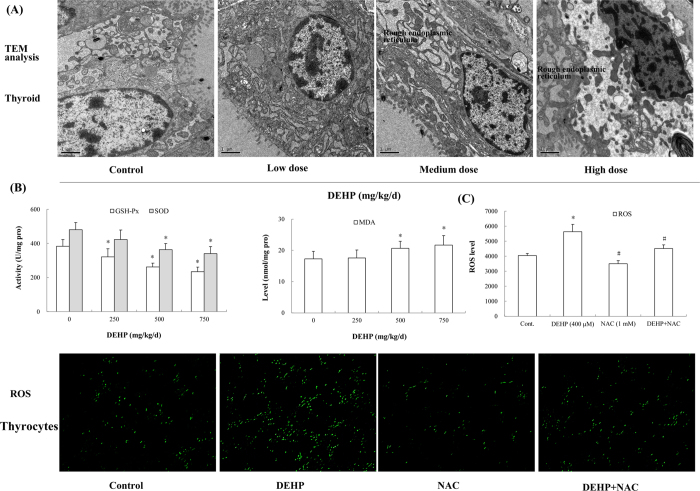
Effects of DEHP on the ultrastructure of rat thyroid, oxidative stress and ROS production. Rats were treated daily with DEHP (0, 250, 500, 750 mg/kg) by gavage for 30 days. Ultrastructural alterations in the thyroid were assessed by TEM analysis. Magnification: X10000. ROS production in cells was evaluated utilizing the oxidant-sensitive probe DCFH-DA. Magnification: X100. (**A**) DEHP caused ultrastructural changes in rat thyroid. (**B**) Antioxidant enzymes, GSH-Px and SOD, were depleted; MDA, the product of lipid peroxidation, was accumulated. (**C**) ROS level in cells was induced by DEHP and was antagonized by NAC. Error bars represent the standard deviation. *P < 0.05, compared with the control. ^#^*P* < 0.05, compared with the DEHP-treated group.

Transmission electron microscope (TEM) analysis further discovered ultrastructural changes in the thyroid of rats exposed to DEHP. Especially in the high-dose group, the nucleus was deformed, the nuclear membrane was shrinked and chromatin was aggregated. Dilation of rough endoplasmic reticulum as well as appearance of vacuoles was also observed (Fig. 2A).

### DEHP caused oxidative stress and ROS production

After DEHP treatment, oxidative stress occurred in rat liver. Compared with the control, the activity of GSH-Px exhibited a dose-dependent decline and that in the low-, medium- and high-dose group was reduced by 16.1% (*P* = 0.007), 31.6% (*P* < 0.001) and 38.9% (*P* < 0.001), respectively. SOD activity also displayed a significantly decreasing trend relative to the control (*P* = 0.001). In contrast, MDA was accumulated and the level was elevated significantly following exposure to DEHP. In the high-dose group, MDA level was enhanced by 25.6% in comparison with the control (Fig. 2B; *P* = 0.007).

In Nthy-ori 3-1 cells, DEHP treatment also significantly induced the production of ROS. ROS level in the DEHP-treated group was upregulated by 39.2% compared with the control (*P* < 0.001). However, antioxidant NAC depressed ROS production caused by DEHP. ROS level in cells treated with DEHP and NAC was declined by 20.0% relative to DEHP-treated cells (Fig. 2C; *P* < 0.001). The green DCF fluorescence also exhibited similar changed characteristics in cells.

### DEHP activated the ERK and Akt pathway *in vivo*

**Figure 3:**
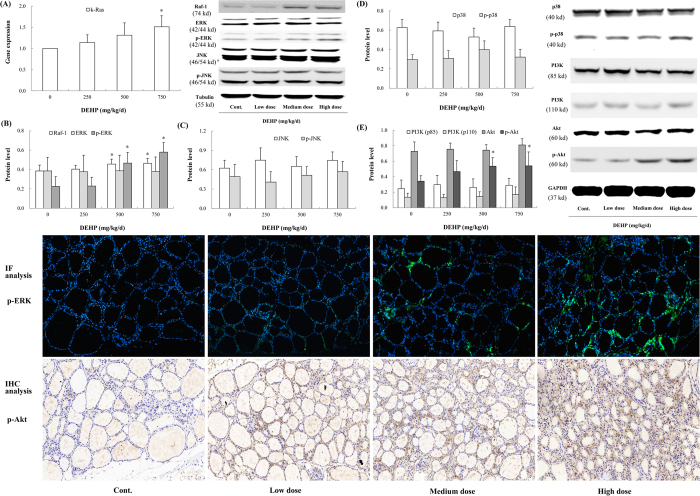
Effects of DEHP on the MAPK and PI3K/Akt pathways *in vivo*. Rats were treated daily with DEHP (0, 250, 500, 750 mg/kg) by gavage for 30 days. p-ERK level in rat thyroid was assessed by IF analysis, and p-Akt by IHC analysis. Green fluorescence and brown indicated positive expressions of p-ERK and p-Akt, respectively. Magnification: X200. (**A**) The gene expression of k-Ras was upregulated by DEHP. (**B**) The ERK pathway was activated, as characterized by induced Raf-1 and p-ERK. (**C**) No significant changes in JNK and p-JNK were observed. (**D**) The p38 pathway was not activated after DEHP exposure. (**E**) The phosphorylation of Akt (Ser473) was significantly elevated, though little changes in PI3K (p85 and p110) were exhibited. Error bars represent the standard deviation. *P < 0.05, compared with the control.

The Ras/Raf-1/ERK pathway was activated after DEHP exposure. The gene expression of k-Ras displayed an increasing trend, with significant effects observed in the high-dose group (Fig. 3A; *P* = 0.029). The protein levels of Raf-1 and p-ERK were also significantly upregulated relative to the control, and that in the high-dose group were elevated by 20.5% (*P* = 0.019) and 111.9% (*P* < 0.001), respectively. IF analysis of p-ERK also demonstrated that the phosphorylation of ERK was induced in rat thyroid. However, DEHP exposure did not affect the protein level of total ERK (Fig. 3B). In addition, the JNK and p38 pathways were not significantly influenced after DEHP treatment (Fig. 3C,D). An intriguing finding was also observed in the present study. DEHP significantly promoted the phosphorylation of Akt (Ser473), which was increased by 57.4% in the high-dose group (*P* = 0.027). IHC analysis also revealed the upregulated p-Akt expression in rat thyroid. However, no significant changes were observed in the protein level of PI3K (p85 and p110) (Fig. 3E).

### DEHP affected protein levels of TSHr and TRHr in rat hypothalamus

**Figure 4:**
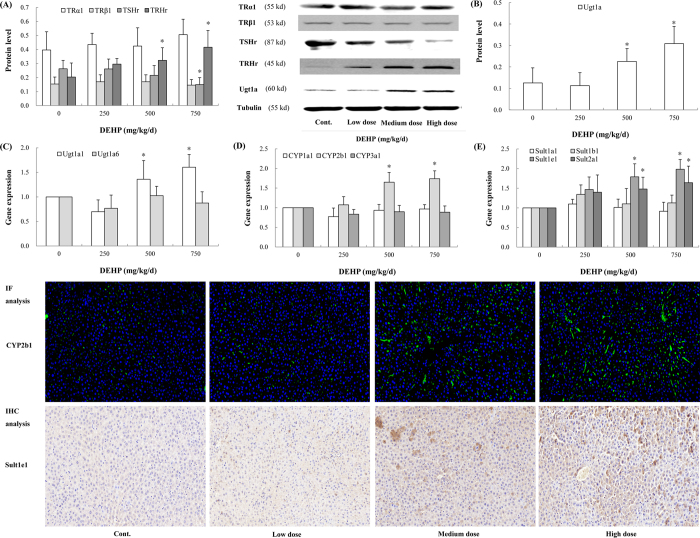
Effects of DEHP on hormone receptors and hepatic enzymes *in vivo*. Rats were treated daily with DEHP (0, 250, 500, 750 mg/kg) by gavage for 30 days. CYP2b1 level in rat thyroid was assessed by IF analysis, and Sult1e1 by IHC analysis. Green fluorescence and brown indicated positive expressions of CYP2b1 and Sult1e1, respectively. Magnification: X200. (**A**) Protein levels of TSHr and TRHr were altered after DEHP treatment. (**B**) Ugt1a protein level was induced following DEHP exposure. (**C**) Ugt1a1 gene expression was induced by DEHP. (**D**) DEHP induced the gene expression of CYP2b1. (**E**) DEHP induced the gene expression of Sult1e1 and Sult2a1. Error bars represent the standard deviation. *P < 0.05, compared with the control.

DEHP exposure significantly suppressed TSHr protein expression. Compared with the control, TSHr level in the high-dose group was declined by 43.0% (*P* = 0.017). In contrast, a dose-dependent increase in the protein level of TRHr was observed. Especially in the high-dose group, TRHr protein level had a 2.0-fold upregulation over the control (*P* = 0.002). However, significant alterations in TRα1 and TRβ1 protein levels were absent from the current study (Fig. 4A).

### DEHP induced expressions of hepatic enzymes

Hepatic enzymes were significantly induced after DEHP exposure. The protein level of Ugt1a exhibited an increasing trend and that in the high-dose group had a 2.5-fold elevation over the control (Fig. 4B; *P* < 0.001). The gene expression of Ugt1a1 in the medium- (*P* = 0.029) and high-dose group (*P* = 0.018) was also higher than that in the control. However, the mRNA expression of Ugt1a6 was not significantly affected after DEHP exposure (Fig. 4C). Moreover, the mRNA level of CYP2b1 was upregulated in a dose-dependent manner, and that in the medium- and high-dose group was increased by 65.0% (*P* = 0.042) and 74.0% (*P* = 0.022) over the control, respectively. IF analysis also indicated that CYP2b1 gene was induced after DEHP treatment. However, DEHP did not significantly induce the gene protein of CYP1a1 and CYP3a1 (Fig. 4D). Furthermore, DEHP significantly induced the mRNA expression of Sult1e1 and Sult2b1. In comparison with the control, a 1.8- (*P* = 0.017) and 2.0-fold (*P* = 0.004) increase in Sult1e1 gene expression was observed in the medium- and high-dose group, respectively (Fig. 4E). IHC analysis of Sult1e1 showed similar findings.

### DEHP activated the ERK and Akt pathway *in vitro*

**Figure 5:**
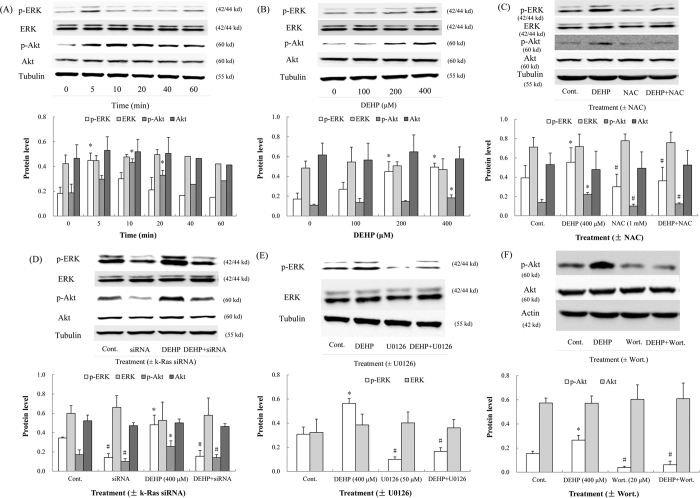
Effects of DEHP on the ERK and Akt pathways *in vitro*. NAC, small interfering RNA (k-Ras siRNA) and inhibitors (U0126 and wortmannin) were utilized in cells. (**A**) DEHP induced the ERK (5 min) and Akt (10 and 20 min) pathways. (**B**) DEHP (400 μM) displayed maximum induced effects on p-ERK and p-Akt. (**C**) NAC antagonized the induced effects of DEHP on the ERK and Akt pathways. (**D**) In k-Ras-silenced cells, p-ERK and p-Akt protein levels were suppressed. (**E**) U0126 inhibited the phosphorylation of ERK. (**F**) The phosphorylation of Akt was inhibited by wortmannin (Wort.). Error bars represent the standard deviation. *P < 0.05, compared with the control. ^#^*P* < 0.05, compared with the DEHP-treated group.

The ERK and Akt pathways were also significantly activated in Nthy-ori 3-1 cells. After treatment with DEHP for 5 min, the phosphorylation of ERK was enhanced (*P* < 0.001); after exposure to DEHP for 10 min, p-Akt protein level was promoted (Fig. 5A; *P* < 0.001). Moreover, p-ERK and p-Akt levels were upregulated in a concentration-dependent manner. Especially in the 400 μM group, p-ERK and p-Akt protein level had a 2.9- (*P* = 0.001) and 1.7-fold (*P* = 0.021) increase relative to the control, respectively (Fig. 5B).

### NAC, siRNA and inhibitors inactivated the ERK and Akt pathways *in vitro*

After treatment with the antioxidant NAC, p-ERK and p-Akt protein levels were reduced significantly. p-ERK (*P* < 0.001) and p-Akt (*P* = 0.038) levels in the cells treated with DEHP and NAC were both lower than that in the cells treated with DEHP (Fig. 5C). To verify the induced effects of k-Ras on the ERK and Akt pathways, knock down of k-Ras gene in Nthy-ori 3-1 cells was performed by k-Ras siRNA. After the transfection with siRNA, protein levels of p-ERK and p-Akt in the cells treated with DEHP and siRNA were reduced by 67.8% (*P* < 0.001) and 44.7% (*P* < 0.001) relative to that in the cells treated with DEHP, respectively (Fig. 5D). The inhibitor U0126 significantly suppressed the level of p-ERK (Fig. 5E; *P* < 0.001). The phosphorylation of Akt was also inhibited by Wort. (Fig. 5F; *P* < 0.001).

### DEHP elevated TRHr level by the Akt pathway

**Figure 6:**
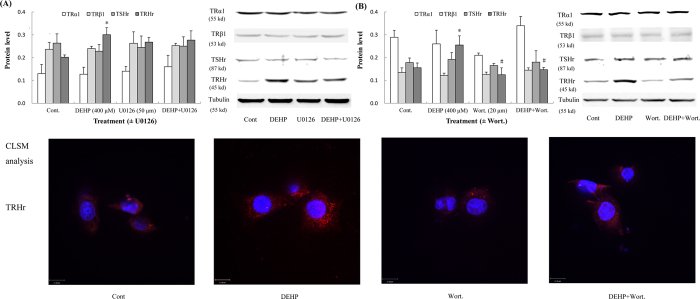
Effects of DEHP on hormone receptors *in vitro*. Inhibitors (U0126 and wortmannin) were utilized in cells. TRHr expression was evaluated by CLSM analysis. Red fluorescence indicated positive expressions of TRHr. Nuclei were stained with DAPI (blue). Magnification: X600. (**A**) TRHr protein level was elevated after treatment with DEHP for 5 min; however, it was not regulated by the ERK pathway. (**B**) TRHr protein level was also upregulated and changed with the status of the Akt pathway (activated or not) after treatment with DEHP for 10 min. No significant alterations in TRα1, TRβ1 and TSHr were observed. Error bars represent the standard deviation. *P < 0.05, compared with the control. ^#^*P* < 0.05, compared with the DEHP-treated group.

After treatment with DEHP *in vitro*, a significant increase in TRHr protein level was observed; however, TRα1, TRβ1 and TSHr levels were not altered in cells. To further elucidate relations between upregulated TRHr and activated signal pathways, inhibitors of the ERK and Akt pathways (U0126 and Wort.) were utilized *in vitro* study. When the ERK pathway was inhibited by U0126, TRHr level was not significantly changed (Fig. 6A). Conversely, when the Akt pathway was suppressed by Wort., TRHr protein level was significantly downregulated in cells treated with DEHP and Wort. (Fig. 6B; *P* = 0.012). Similar results were also observed by CLSM analysis, and TRHr protein expression was lower in the presence of DEHP and Wort.

## Discussion

The present study explored roles of signaling pathways and hepatic enzymes in DEHP-mediated perturbation of TH homeostasis. The findings indicate that DEHP could decline circulating TH levels via the Ras/Akt/TRHr pathway. Likewise, DEHP-induced hepatic enzymes contribute to the downregulation of THs.

Numerous studies have shown that DEHP and its metabolites could induce oxidative stress in biological systems^6^,^19^,^20^. Oxidative stress is induced by an imbalance between the production and elimination of ROS in the system, and will occur when ROS production surpasses the body’s natural antioxidant defense mechanisms, causing antioxidant enzyme depletion, lipid peroxidation, DNA damage, etc. Moreover, several studies have suggested that multiple signaling pathways such as the MAPK and PI3K/Akt pathways could be affected and regulated by oxidative stress in various cells and animal models^17^,^21^. In the current study, we found the development of oxidative stress after DEHP treatment, as characterized by ROS production in thyrocytes, GSH-Px and SOD depletion, as well as MDA accumulation in rat liver. DEHP-induced oxidative stress further activated the Ras/Raf-1/ERK pathway, and the gene expression of k-Ras and protein levels of Raf-1 and p-ERK were significantly upregulated. Though the activated sign of PI3K was absent from the current study, the phosphorylation of Akt was significantly induced in the HPT axis. To figure out how DEHP activated the Akt pathway, the siRNA of k-Ras were utilized *in vitro* study and results indicated that k-Ras could be an upstream signal to induce the Akt pathway in the absence of PI3K stimulation. The finding is in agreement with those previous researches. Ras, a small GTP-binding protein, is an upstream activator of several signaling pathways including ERK and Akt^22^. Statins suppressed p-ERK and p-Akt levels by inhibiting the membrane localization of k-Ras^23^. Another study also observed that inhibition of RAS activity resulted in significant decreases in the phosphorylation of ERK and AKT, eventually leading to the apoptosis of human meningioma cells^24^. To further verify the involvement of oxidative stress in ERK and Akt activation, the antioxidant NAC was used *in vitro* study. It was found that ROS production in thyrocytes was antagonized after co-incubation with DEHP and NAC, followed by the suppression of p-ERK and p-Akt levels. These results indicate that DEHP-caused oxidative stress exerts activated effects on the ERK and Akt pathways. Nevertheless, DEHP did not induce the JNK and p38 pathways in the current study, which was inconsistent with other studies. Oh *et al*.^25^ found DEHP could stimulate the expression of IgE, COX-2, and IL-4 through the p38 pathway in ICR mice. And in human umbilical vein endothelial cells, the activation of ERK and p38 pathways contributed to DEHP-induced ICAM-1 and IL-8 expression^26^. We postulate that this difference maybe rest in diverse biological functions of signaling pathways, as well as disparate research objects.

Thyroid hormone-related receptors, including TRα1, TRβ1, TSHr and TRHr, are important elements in regulating the synthesis and release of THs. TRα1 and TRβ1, which are nuclear transcription factors, have extensive expressions in almost all tissues. TSHr and TRHr are both the G protein-coupled receptors with seven transmembrane domains. TSHr locates on the basal surface of thyroid follicular cells while TRHr is distributed throughout the central and peripheral nervous systems^27^. In the HPT axis, TRH produced by the hypothalamus binds to the TRHr, thereby promoting the release of TSH; TSH produced by the pituitary binds to the TSHr, thereby stimulating the release of THs; THs synthesized by the thyroid bind to TRα and TRβ, then modulating TH homeostasis via the negative feedback system of the HPT axis. *In vivo* study, declined TSHr and elevated TRHr protein levels were observed, whereas TRα1 and TRβ1 expressions were not significantly influenced after DEHP exposure. *In vitro* study, TRHr protein level was also upregulated following treatment with DEHP in thyrocytes. To further elucidate relations between enhanced TRHr and activated ERK and Akt pathways, inhibitors (U0126 and Wort.) were also utilized *in vitro* study. When the Akt pathway was activated, TRHr expression was upregulated; when the Akt pathway was inhibited by Wort., TRHr level was downregulated subsequently. However, TRHr level was not affected by the status of the ERK pathway (activated or not). It is known that aberrant expressions of hormone receptors will perturb the HPT axis, leading to the abnormality of hormone signal transduction. In the present study, T3 and T4 levels in serum were decreased; however, TSH and TRH levels were not upregulated to compensate the decline in THs, indicating the impairment of the negative feedback system of HPT axis. The insensitivity of TSH as a marker of HPT axis and TH imbalance is consistent with findings in studies on other endocrine disruptors^17^,^28^. Meanwhile, it should be noted that significant changes in TSHr level were not observed *in vitro* study, suggesting that TSHr is not involved in Ras/Akt-mediated disturbance of HPT axis. Above findings demonstrate that the Ras/Akt pathway disturbs the HPT axis via modulating TRHr expression, further influencing the TH homeostasis.

Thyroid hormone levels are modulated not only by synthesis and secretion but also by metabolism and clearance. Therefore, the hepatic-endocrine axis is another important component in TH homeostasis^29^. Hereon, our current study suggests that the induction of hepatic enzymes by DEHP is another vital mechanism for the disruption of TH homeostasis. THs are metabolized predominantly in the liver and are excreted into bile. Hepatic CYP450s that are heme-containing drug-metabolizing enzymes with oxidase activity are found at high levels in the liver. These hepatic microsomal phase I enzymes are responsible for the biotransformation and metabolism of various endogenous compounds, including THs. In the current study, CYP2b1 gene was significantly induced and a 1.7-fold increase was observed after DEHP exposure. In addition, the catabolism and excretion of THs is also catalyzed by the hepatic microsomal UGTs, which are hepatic microsomal phase II enzyme and are found mostly in the endoplasmic reticulum of the liver. More specifically, UGTs catalyze conjugation of THs with glucuronic acid to elevate the water solubility and excretion through the bile and urine^30^. In our study, Ugt1a1 was also significantly induced, as characterized by upregulated gene and protein expressions. Our results are in accordance with many previous studies in similar endocrine disruptors. The observed degree of TH reduction after DE-71 exposure corresponded with induction of hepatic enzymes, CYP1a1, CYP2b1/2, CYP3a1 and UGTs^31^. Fisher *et al*.^32^ observed that hepatic CYP1a1 and UGT activities were induced in PCB126-caused perturbation in the HPT axis. After exposure to p,p’-DDE, induced hepatic enzymes also contributed to the decline of THs in rat serum^33^. Moreover, sulfation is also an important pathway of TH metabolism via elevating their hydrophilicity and subsequent biliary and urinary excretion. Sulfate conjugation is catalyzed by various sulfotransferases (Sults) present in cytosol, which are widely expressed in hepatic tissue and in metabolically active or hormonally responsive extrahepatic tissues^34^. In the current study, expressions of Sult1e1 and Sult2a1 were significantly induced following DEHP exposure, contributing to the degradation and inactivation of T3 and T4; whereas significant alterations in Sult1a1 and Sult1b1 mRNA expressions were absent from this study. Szabo *et al*.^31^ also reported that Sult1b1 expression was elevated, whereas Sult1a1 and Sult1c1 levels were not affected in DE-71-causing disruption of TH homeostasis. We analyze that diverse biological characteristics of isoforms, such as that iodothyronines are good substrates for Sult1e1^35^, may explain the different induced effects.

**Figure 7:**
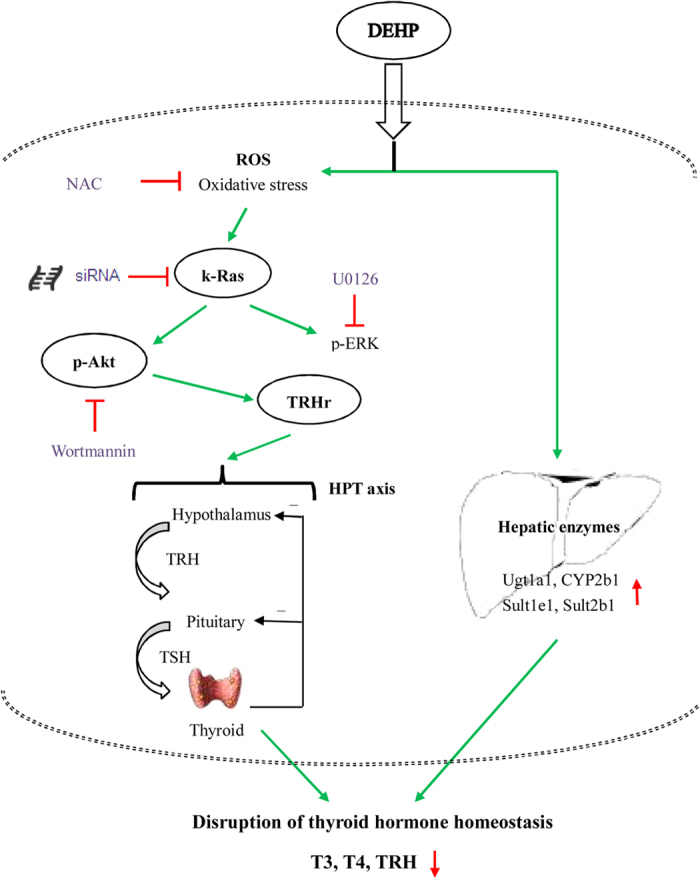
Effects of DEHP on thyroid hormone homeostasis. The potential mechanisms included the activated Ras/Akt/TRHr pathway and induced hepatic enzymes.

In conclusion, our current study demonstrates that DEHP has thyroid-disrupting effects and can decline circulating TH levels in serum though activating the Ras/Akt/TRHr pathway and inducing hepatic enzymes (Fig. 7), and this study will contribute to a better understanding of thyrotoxicity of phthalates.

## Additional Information

**How to cite this article**: Ye, H. *et al*. Di2-ethylhexyl phthalate disrupts thyroid hormone homeostasis through activating the Ras/Akt/TRHr pathway and inducing hepatic enzymes. *Sci. Rep.* **7**, 40153; doi: 10.1038/srep40153 (2017).

**Publisher's note:** Springer Nature remains neutral with regard to jurisdictional claims in published maps and institutional affiliations.

## Figures and Tables

**Table 1:** The primer sequences used in the present study.

Primer	Type	Primer Sequence	GenBank
k-Ras	Forward	GCGTAGGCAAGAGTGCCTTGA	NM_031515.3
	Reverse	GACCTGCTGTGTCGAGAATATCCA	
Ugt1a1	Forward	TTGGTGGGATAAACTGCCTTCA	NM_012683.2
	Reverse	GAATTCTGCCCAAAGCCTCA	
Ugt1a6	Forward	CAGTCATGCCCAACATGATCTTC	NM_001039691.2
	Reverse	TCTCCGGAGGCGTTGACATA	
CYP1a1	Forward	ATGAGTTTGGGGAGGTTACTGGT	NM_012540.2
	Reverse	ACTTCTTATTCAAGTCCTTGAAGGCA	
CYP2b1	Forward	TGAGAACCTCATGATCTCCCTGC	NM_001134844.1
	Reverse	AGGAAACCATAGCGGAGTGTGG	
CYP3a1	Forward	CTCTTCACCGTGATCCACAGCACT	NM_013105.2
	Reverse	ATGCTGCCCTTGTTCTCCTTGC	
Sult1a1	Forward	TTCGCAACGCCTACACAAAGA	NM_031834.1
	Reverse	TCACATGCACTAGCGGTGGAC	
Sult1b1	Forward	GCCCACAGAAATAATGGATCACAG	NM_022513.2
	Reverse	GTGCAGAACTCAAGTGTTGTTCCAG	
Sult1e1	Forward	GTGGTGCAATTTGAAGTGAACTGA	NM_012883.1
	Reverse	ATCTGGCCTTGCCAAGAATG	
Sult2a1	Forward	AGAAGCCAGACTCACTGGGAACTTA	NM_131903.1
	Reverse	CAGGCACGGATGTGCTCAA	
β-Actin	Forward	GGAGATTACTGCCCTGGCTCCTA	NM_031144.2
	Reverse	GACTCATCGTACTCCTGCTTGCTG	

**Table 2:** DEHP increased the liver weight of rats.

DEHP (mg/kg/d)	Body wt (g)	Body wt gain (g)	Liver wt (g)	Relative liver wt (%)
0	171.02 ± 21.7	131.67 ± 17.1	7.61 ± 1.0	4.46 ± 0.4
250	185.82 ± 17.9	144.27 ± 14.2	9.62 ± 1.1*	5.18 ± 0.3*
500	166.13 ± 16.7	124.85 ± 19.4	9.85 ± 0.9*	5.93 ± 0.2*
750	176.03 ± 24.1	131.80 ± 24.3	10.8 ± 2.4*	6.06 ± 0.6*

*Note.* Values were reported as mean ± SD. Relative liver weight is % body weight; *A difference at *P* < 0.05 was considered statistically significant.
